# Two sexually compatible monokaryons from a heterokaryotic *Lentinula edodes* strain respond differently to heat stress

**DOI:** 10.3389/fmicb.2025.1522075

**Published:** 2025-02-12

**Authors:** Yuan Guo, Wenyu Jiao, Yajie Zhang, Meiting Tan, Qi Gao, Yu Liu, Shouxian Wang

**Affiliations:** ^1^Beijing Engineering Research Center for Edible Mushroom, Institute of Plant Protection, Beijing Academy of Agriculture and Forestry Sciences, Beijing, China; ^2^College of Life Sciences and Technology, Mudanjiang Normal University, Mudanjiang, China; ^3^College of Plant Science and Technology, Beijing University of Agriculture, Beijing, China; ^4^College of Agriculture and Food Engineering, Baise University, Baise, China

**Keywords:** *Lentinula edodes*, heat stress response, metabolomics, transcriptomics, multi-omics integration

## Abstract

**Background:**

Despite the extensive research conducted on heat responses of *Lentinula edodes* heterokaryotic cells, the responses of the two sexually compatible monokaryons to heat stress (HS) remain largely unknown.

**Methods:**

To bridge this gap, we examined the nucleus-specific (SP3 and SP30) heat resistant mechanisms using an integrated physiological, metabolomic and transcriptomic approach.

**Results:**

The results showed that HS elicited the boost of ROS and hampered mycelium growth for both monokaryons. Metabolome and transcriptome analysis demonstrated that the two sexually compatible monokaryons responded differently to HS. For SP3, the differentially expressed genes (DEGs) were significantly enriched in Mitogen-Activated Protein Kinase (MAPK) signaling, cell cycle and sugar metabolism, whereas those DEGs for SP30 were enriched in glyoxylate and dicarboxylate metabolism, and protein processing. The differentially accumulated metabolites (DAMs) of both strains were enriched in the glycerophospholipid metabolism, alpha-linolenic acid metabolism, biosynthesis of cofactors, etc, but were regulated differently in each strain. The enriched KEGG pathways for SP3 tend to be downregulated, whereas those in SP30 exhibited a contrary trend. The genes in MAPK signaling pathway were associated with the glycerophospholipid metabolism in SP3, but not in SP30. Omics-integration analysis revealed distinguishing regulatory networks and identified completely different hub genes for the two strains.

**Discussion:**

Our findings revealed, for the first time, the different heat-resistance mechanisms of the two compatible nuclei and provided candidate metabolites, responsive genes and regulatory pathways for further experimental validation.

## 1 Introduction

*Lentinula edodes*, known as xianggu in China and shiitake in Japan, is the top 2 species cultivated and consumed worldwide. It constitutes 26% of the world’s total mushroom production (Singh et al., 2020). Shiitake is recognized for its unique flavor, nutritional value and medical properties. It contains versatile bioactive compounds, including polysaccharides, lentinan, ergosterol, nucleic acid derivatives, water–soluble lignin, eritadenine, etc., conferring it promising pharmacology effects comprising antitumor, immunomodulatory, hypocholesterolemic, antibacterial, antifungal, anti–inflammatory and antioxidant properties (Ahmad et al., 2023; Xu et al., 2024). In China, shiitake has been used in traditional medicine for more than 2000 years (Zhang et al., 2023).

Shiitake is broadly distributed across subtropical to temperate regions of the northern hemisphere. Its cultivation happens worldwide, while most of its production is from east Aisa such as China, Japan and South Korea (Menolli et al., 2022; Sierra-Patev et al., 2023). The optimal temperature for the growth of *L. edodes* mycelia and fruiting is 24–27°C and 8–20°C, respectively (Zhao et al., 2019). For shiitake production, heat stress is one of the major environmental constrains, exerting a deleterious effect on its productivity. Temperatures that exceed 25°C can result in a highly reduced commercial value of the shiitake fruiting body, for instance the small fruiting body, thin cap, and less open umbrella (Yang et al., 2024). Temperatures higher than 30°C severely inhibit mycelia growth and fruiting body formation processes (Shen et al., 2017). Heat stress has caused significant yield loss and quality deterioration of shiitake production in almost all its production areas. Unfortunately, extreme heat events are expected to intensify due to the ongoing global climate change (González-García et al., 2023).

Extensive literatures have documented the impacts of HS on mycelial development of shiitake, ranging from the morphological changes to metabolic reprogramming and molecular regulation. Morphologically, heat stress markedly hampers mycelial growth and weakens the mycelial regeneration when HS is removed (Guo et al., 2023; Zhang et al., 2023). Using microscope and SEM (scanning electron microscope), damages to the cell surface and loss of cytoplasmic inclusions could be observed upon exposure to heat (Wang et al., 2018). *L. edodes* mycelia, moreover, can respond to heat stress on physiological level by activating the antioxidant system to alleviate the damage to mycelial cell (Luo et al., 2021). In general, heat stress can induce an elevation in the reactive oxygen species (ROS) level, which subsequently triggers lipid peroxidation in the cell membrane. As a result, enzymatic (superoxide dismutase, catalase, peroxidase, etc.) and non-enzymatic (reduced glutathione, trehalose, indoleacetic acid, linoleic acid, etc.) antioxidants are activated to scavenge excess ROS to relieve cell injuries (Wang et al., 2018; Wang et al., 2020; Guo et al., 2023; Zhang et al., 2023; Yang et al., 2024). The omics-based approaches offer powerful strategies to gain a global landscape of mechanisms by which shiitake copes with heat stress. Using a combined proteomic and transcriptomic approach, Wang et al. (2018) found that the heat shock proteins (HSPs) and IAA were functioning to the heat resistance of shiitake mycelium. Using transcriptomic techniques, Xu et al. (2022) investigated the mechanism by which 2,4-dichlorophenoxyacetic acid (2,4-D) improved the thermotolerance of *L. edodes*.

Probably because the dikaryotic stage represents the longest period in the life cycle of shiitake, all the literature dealing with its heat-resistance mechanisms has focused on the dikaryotic mycelia. Undoubtedly, those dikaryon-based heat-resistance research is crucial to understanding how *L. edodes* against thermostress. Nonetheless, with the aim of breeding for the superior heat-resistant varieties, the importance of the dikaryon-based documents could be compromised, because current shiitake breeding practices are basically using monokaryons as parents. However, so far, how the monokaryotic mycelia cope with heat stress at physiological and molecular levels remain largely unknown. Recent genome sequencing studies of the two sexually compatible monokaryons of a *L. edodes* strain showed that their genomic structures harbored pronounced differences (Gao et al., 2022; Shen et al., 2024), implying that the two haploid nuclei might handle high temperature differently. More recently, Yoo et al. (2024) found that the monokaryons from two *L. edodes* strain characterized by thermo-tolerance and thermo-sensitivity may have different thermotolerance by a comparative genomic investigation. Unfortunately, in this study physiological and molecular evidence are lacking.

To fill the gap of the monokaryon-specific mechanisms in response to heat stress, we investigated the morphological, physiological, metabolic and transcriptomic changes of two sexually compatible monokaryons from a widely cultivated *L. edodes* strain under normal and high temperature conditions. Our results demonstrate, for the first time, that the two sexually compatible monokaryons respond differently to HS, deepening our understanding of the heat-resistance mechanism of *L. edodes*.

## 2 Materials and methods

### 2.1 Fungal strains and cultivation

The two sexually compatible monokaryotic strains SP3 and SP30 were obtained from the dikaryotic *L. edodes* strain JZB2102217, using protoplasting method as described by Gao et al. (2022). The strain JZB2102217 is a widely used high-temperature type cultivar in northern China. Mycelia from original cultures were punched out using a cork borer (1 cm diameter), and then inoculated in petri dishes (10 cm diameter) containing 35 mL of potato dextrose agar (PDA) medium as described previously (Gao et al., 2022; Guo et al., 2023). Prior to inoculation, a sterilized cellophane membrane was placed on the surface of the PDA medium for efficient collection of the mycelium samples. The two strains were cultivated in a growth incubator at 25°C and in permanent darkness. After 6 days of growth at 25°C, the fungal cultures were divided into control and treatment groups, in which the latter were subjected to heat exposure at 37°C for 12 h.

### 2.2 Measurement of mycelial growth

Fungal colony diameter was measured every two days once the inoculant was germinated. The inhibition rate was calculated as follows:


$$\mathrm{growth}\;\mathrm{inhibition}\;\mathrm{rate}=\frac{G1-G2}{G2}\times 100\%$$

where G1 and G2 denote the growth rate of the fungal colony after and before heat treatment. Growth rate was calculated according to the diameter change during the period of growth before and after heat treatment (mm/d).

### 2.3 Detection of reactive oxygen species and malondialdehyde content

Reactive oxygen species (ROS) production was measured according to the previously described method (Ren et al., 2017; Guo et al., 2023) with modifications. Briefly, for 2′,7′-dichlorodihydrofluorescein diacetate (DCFH-DA) staining, sterile glass coverslips were obliquely inserted into the petri dishes after the inoculation of fungal blocks, allowing the fungal mycelium to grow later. When hyphae grew on the coverslips, the coverslips with hyphae were incubated in 10 μmol/L of DCHF-DA (Solarbio Life Sciences, Beijing, China) phosphate-buffered saline (PBS) solution for 25 min under dark conditions for ROS visualization. After staining, ROS production was visualized using a fluorescence microscope (Olympus, IX71, Tokyo, Japan). For nitroblue tetrazolium (NBT, Beyotime Biotechnology, Beijing, China) staining, mycelial pellets were incubated in 0.5 μg/mL NBT aqueous solution for 2 h, and the intensity was monitored under a light microscope. The hydrogen peroxide (H_2_O_2_) content was measured using assay kit following the manufacturer’s instruction (Solarbio Life Sciences, Beijing, China). For measurement of MDA content, 0.1 g of fresh mycelium samples were used for MDA extraction following the manufacture’s instruction (Solarbio Life Sciences, Beijing, China).

### 2.4 RNA isolation, sequencing, differential expression analysis and functional annotation

Total RNA was extracted from mycelia of *L. edodes* (five replicates) using TRIzol® Reagent according to the manufacturer’s instruction (Invitrogen, Carlsbad, CA, USA). The RNA quantity determination, libraries construction, and Illumina (Illumina, San Diego, CA, USA) sequencing were performed according to the protocols described by Guo et al. (2023). The raw paired end reads were trimmed, and quality controlled by SeqPrep (https://github.com/jstjohn/SeqPrep) and Sickle (https://github.com/najoshi/sickle) with default parameters. Clean reads were separately aligned to reference genome with orientation mode using HISAT2 (http://ccb.jhu.edu/software/hisat2/index.shtml) software (Kim et al., 2015). The reference genome sequence data reported in this paper have been deposited in the Genome Warehouse (Chen et al., 2021) in National Genomics Data Center, Beijing Institute of Genomics, Chinese Academy of Sciences / China National Center for Bioinformation, under accession number GWHBGWX00000000 that is publicly accessible at https://ngdc.cncb.ac.cn/gwh/Assembly/23974/show. The mapped reads of each sample were assembled by StringTie in a reference-based approach (Pertea et al., 2015).

Gene expression levels (fragments per kilobases per million reads) were calculated using the RESM software (http://deweylab.biostat.wisc.edu/rsem/) (Li and Dewey, 2011). Differentially expressed genes (DEGs) were identified using the DESeq2 (Love et al., 2014) with the following general criteria: |log2FC| > 1 and adjusted *p* value (padjust) ≤ 0.05. The padjust was calculated using the BH (FDR correction with Benjamini/Hochberg) methods (Benjamini and Hochberg, 1995). Transcripts were annotated using the functional database including Gene Ontology (GO), Kyoto Encyclopedia of Genes and Genomes (KEGG), NCBI non-redundant protein sequences (NR), Swiss-Prot, Protein family (Pfam), and Evolutionary Genealogy of Genes: Non-supervised Orthologous Groups (EggNOG) (Zhang et al., 2024).

### 2.5 Non-targeted metabolomics analysis

Fifty mg of mycelium was used for metabolite measurements using an ultra-high performance liquid chromatography (UHPLC) system (Thermo Electron Corporation, San Jose, CA, USA), coupled with Thermo UHPLC-Q Exactive Mass Spectrometer (MS/MS) (Thermo Electron Corporation, San Jose, CA, USA). Mycelium sample preparation, metabolite extraction, chromatographical measurement, and raw chromatographic data analysis were performed according to the previously described protocol (Guo et al., 2023). Metabolic features detected at least 80% in any set of samples were retained. After filtering, minimum metabolite values were imputed for specific samples in which the metabolite levels fell below the lower limit of quantitation and each metabolic features were normalized by sum. The internal standard (2-Chloro-L-Phenylalanine, HPLC hyper grade, Merck, Darmstadt, Germany, 0.02 mg/mL) was used for quantitative determination of metabolites. Metabolic features which the relative standard deviation (RSD) of QC > 30% were discarded. Compounds were identified by comparison of accurate mass, MS/MS fragments spectra and isotope ratio difference against biochemical databases Human metabolome database (HMDB) (http://www.hmdb.ca/) and Metlin database (https://metlin.scripps.edu/). The mass tolerance between the measured m/z values and the exact mass of the components of interest was ±10 ppm. Mass features without MS/MS spectra were tentatively annotated on MS1 level using 5.0 mDa tolerance. The metabolite abundances were quantified according to their peak areas.

Differentially accumulated metabolites (DAMs) (VIP in OPLSDA model ≥1, false discovery rate (FDR) ≤ 0.05, Fold change ≥ 1) between groups were mapped into biochemical pathways through metabolic enrichment and pathway analysis based on database search (KEGG, http://www.genome.jp/kegg/). To identify statistically significantly enriched pathway, scipy.stats (Python packages) (https://docs.scipy.org/doc/scipy/) was used with Fisher’s exact test.

### 2.6 Statistical analysis

Principal component analysis (PCA) and orthogonal partial least square regression discriminant analysis (OPLS-DA) were performed to examine overall variations of the metabolomics and transcriptomic data. PCA was conducted using the “FactoMineR” (Lê et al., 2008) and “factoextra” (Kassambara and Mundt, 2017) package in R (version 4.4.1). OPLS-DA was performed using the SIMCA 14.1 (Umetrics, Umeå, Sweden). The robustness of OPLS-DA models was assessed by seven-fold cross-validation. The reliability and accuracy of the predictive models was assessed by analysis of variance testing of cross-validated predictive residuals (CV-ANOVA), R2 and Q2metrics. The *p* value was estimated with paired student’s *t*-test on single dimensional statistical analysis. To test the overall associations between DEGs and DAMs, we performed Procrustes analysis using the R package “vegan” (version 2.7–0) (Dixon, 2003). Data were logarithmically (log10) transformed, centered, and pareto scaled prior to multivariate analyses. For integration and visualization of transcriptome × metabolome associations, the sparse partial least squares (sPLS) regression method (Lê Cao et al., 2008) was implemented using the R package “mixOmics” (Rohart et al., 2017).

## 3 Results

### 3.1 Thermostress triggered ROS production and inhibited mycelial growth of the two monokaryons

**Figure 1:**
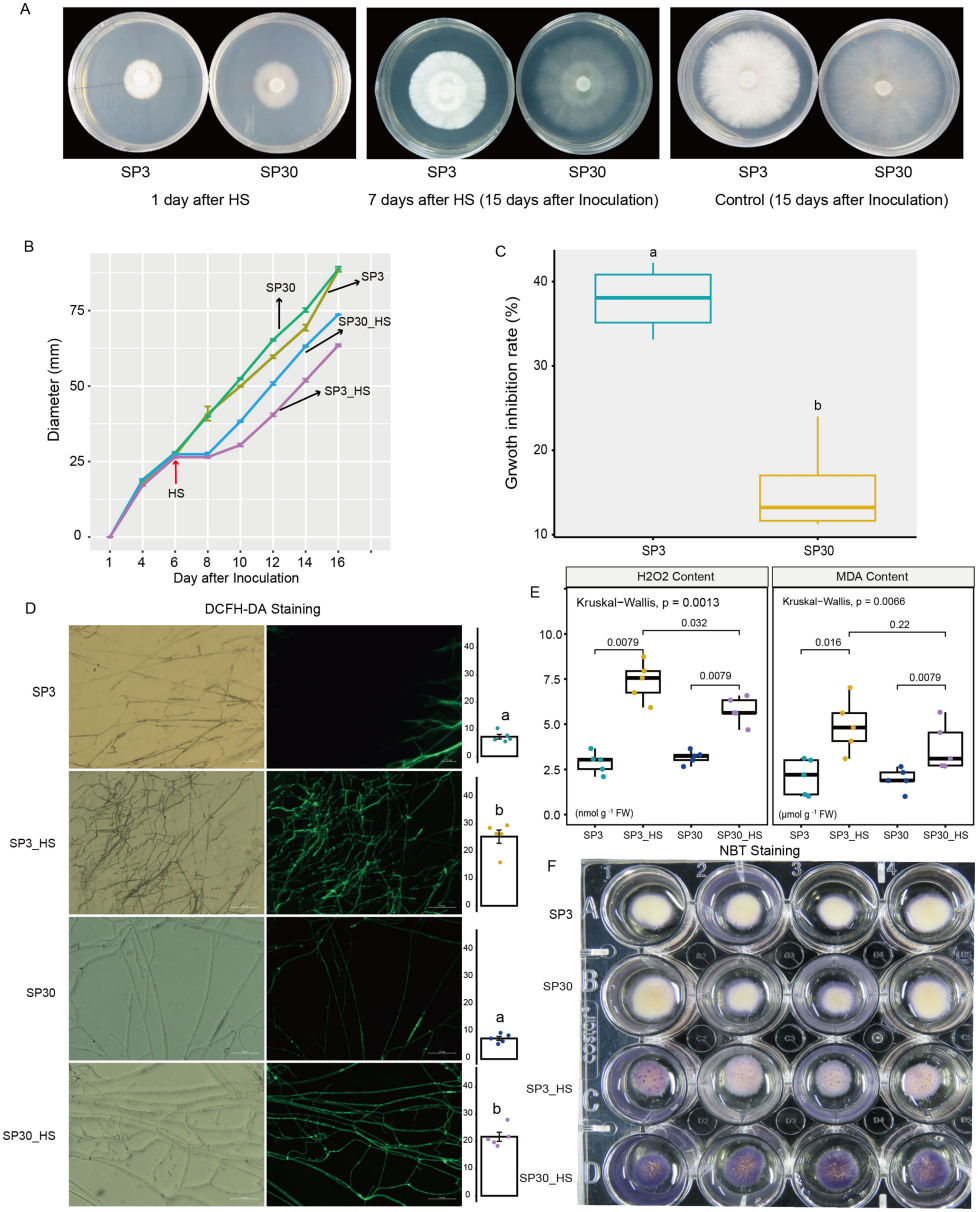
Morphological and physiological responses of SP3 and SP30 strains under control (25°C) and heat stress (HS, 37°C) conditions. The fungal hyphae of two strains were able to recover growth at 2 days after HS. **(A)** The morphology of SP3 and SP30 cultures at 1st and 15th days after inoculation (7th days after HS). **(B)** The fungal colony diameters of different strains. HS was conducted on the 6th day after inoculation. **(C)** The growth rate and growth inhibition rate of two strains in response to HS. **(D)** The ROS staining (bar = 50 μm) and mean fluorescence intensity. Different letters indicate significant differences between groups (ANOVA, *p* < 0.05,). Data are represented as mean ± se (*n* = 5). **(E)** The H_2_O_2_ and MDA contents of the two strains with and without HS. The values above the boxes indicate the *p* value of Kruskal-Wallis test. **(F)** NBT staining of the two strains with and without HS.

We first examined the heat stress (HS) responses of the two sexually compatible monokaryons on the morphological and physiological levels (Figure 1). Results demonstrated that HS markedly inhibited the growth rate of mycelium for both monokaryons. When HS was removed, the regenerated mycelium appeared weak compared to the old mycelium formed prior to HS exposure (Figures 1A,B). Based on the average growth rate of the two monokaryons before and after HS, we found that the growth inhibition rate of SP3 strains was significantly higher than that of SP30 strains (Figure 1C). This suggested that the growth of SP3 might be more inhibited than SP30 subjected to HS. DCFH-DA and NBT staining showed that ROS contents were comparable forboth strains at room temperature, whereas were significantly increased when challenged with HS (Figures 1D,F). We also measured the H_2_O_2_ content by enzymatic assay, which showed consistent results with the ROS staining assay. The MDA production, a marker of the cell membrane lipid peroxidation, was increased significantly under HS (Figure 1E).

### 3.2 The two monokaryons showed distinct metabolic profiles under normal and HS conditions

Non-targeted metabolomics was applied to examine the metabolic variations of mycelium of the two monokaryons. In total, 1,122 metabolites were detected (Supplementary Table 1). Based on the HMDB superclass, 33.59% of those compounds were lipids and lipid-like molecules, and 23.33% of those were organic acids and derivatives (Supplementary Figure 1A). KEGG functional annotation analysis demonstrated that most of those compounds were related to the category of metabolism, followed by the environmental information processing and genetic information processing (Supplementary Figure 1B).

**Figure 2:**
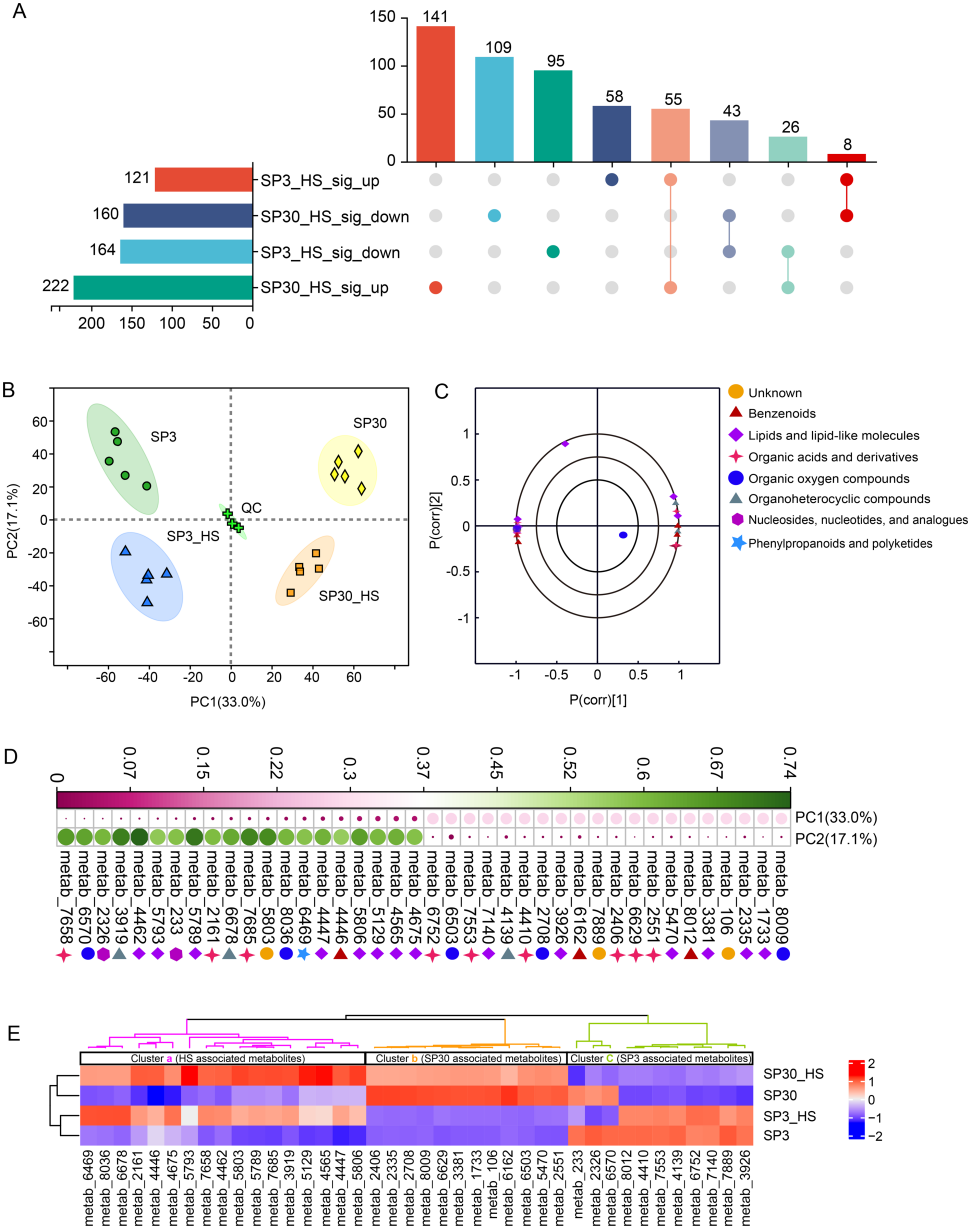
Metabolites detected from SP3 and SP30 strains under control (25°C) and heat stress (HS, 37°C) conditions. **(A)** The Venn analysis for the differentially accumulated metabolites (DAMs). The DAMs were identified according to the following criteria: false discovery rate (FDR) ≤ 0.05, variable influence on projection (VIP) ≥ 1 in OPLS-DA model, fold change ≥ 1. **(B)** PCA score plot generated using all the metabolites. **(C)** PCA loading plot showed the top 20 metabolites contributed to the PCA model. **(D)** Contributions of the top 20 metabolites contributing to the PC1 and PC2. **(E)** Heatmap analysis of the top 20 metabolites for PC1 and PC2. The dendrograms show the hierarchical cluster analysis for the two strains.

The upset plot showed that 285 and 382 differentially accumulated metabolites (DAMs) were found for SP3 and SP30 subjected to HS, respectively. The number of the up-regulated metabolites in SP30 was 1.8 times higher than that in SP3. There were 58 and 141 DAMs were specifically up-regulated for SP3 and SP30 under HS, respectively (Figure 2A). PCA revealed that the metabolic profiles of the two strains exhibited distinct differences and were also distinguishable from those exposed to HS. Interestingly, the first component (PC1), which explained 33.0% of the total variation, could explain the differences resulting from the two strains, whereas the second component (PC2), which explained 17.1% of the total variation, could explain the variation derived from the HS treatment (Figure 2B). The loading plot depicted the top 20 compounds contributed to the PCA model (Figure 2C). Figure 2D demonstrated the top 20 metabolites contributed to PC1 and PC2, respectively. With the heatmap analysis we could observe that those metabolites could classify into 3 clusters (a, b, c). The metabolites in cluster “a” were clearly related to the HS condition, whereas the cluster “b” and “c” were associated with monokaryon SP30 and SP3, respectively (Figure 2E).

### 3.3 The two monokaryons showed distinct transcriptomic profiles under normal and HS conditions

To identify genes and corresponding functional pathways associated with HS for the two monokaryons, we performed a comparative transcriptomics analysis. A total of 129.13 Gb clean data was obtained with a high score of quality (Q30 rate > 93.58%) (Supplementary Table 2). The average mapping rate was 89.96%, and more than 84% of genes were coding sequencing for all samples (Supplementary Tables 3, 4). The functional annotations could be found in Supplementary Table 5.

**Figure 3:**
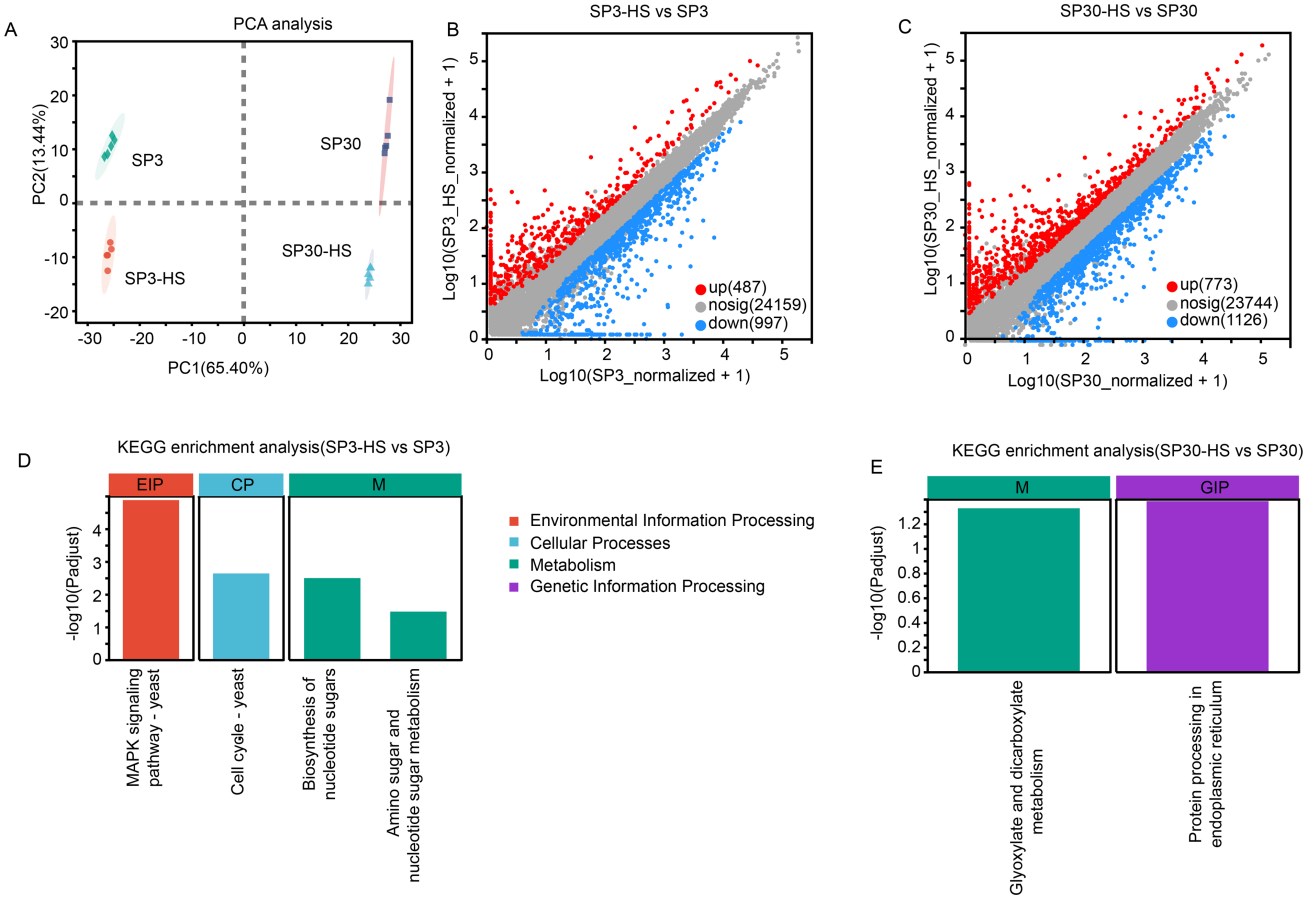
Transcriptome analysis of SP3 and SP30 in response to heat stress. **(A)** Score plot of principal component analysis. **(B,C)** Scatter plots of all expressed genes for SP30 and SP3 under HS, respectively. X-axis and Y-axis represent log10 value of gene expression. Blue dots represent down-regulated genes, red dots denote up-regulated genes, and grey dots denote genes with no significant differences. **(D,E)** KEGG enrichment analysis of the differentially expressed genes for SP3 and SP30 under HS, respectively.

In consistent with the metabolomic data, the transcriptomic profiles of the two monokaryons showed distinct patterns under normal and HS conditions. The first component (PC1), which explained 65.4% of the total variation, could explain the differences resulting from the two strains, while the second component (PC1), which explained 13.44% of the total variation, could explain the variation resulting from the HS treatment (Figure 3A). There were 1,484 and 1899 DEGs identified from SP3 and SP30 in response to HS, respectively. The number of both the up and down-regulated genes in SP30 was higher than that in SP3 in response to HS, particularly for the up-regulated genes in SP30, which were 1.6 times higher than those in SP3 (Figures 3B,C). Using the KEGG enrichment analysis, we observed that the DEGs in SP3 subjected to HS were significantly enriched in environmental information processing (MAPK signalling pathways), cell cycle, and metabolism (biosynthesis of nucleotide sugar, and amino sugar and nucleotide sugar metabolism), whereas those in SP30 were glyoxylate and dicarboxylate metabolism, and genetic information processing (protein processing in endoplasmic reticulum) (Figures 3D,E).

### 3.4 Identification of the key metabolic pathway of the two monokaryons in response to HS

**Figure 4:**
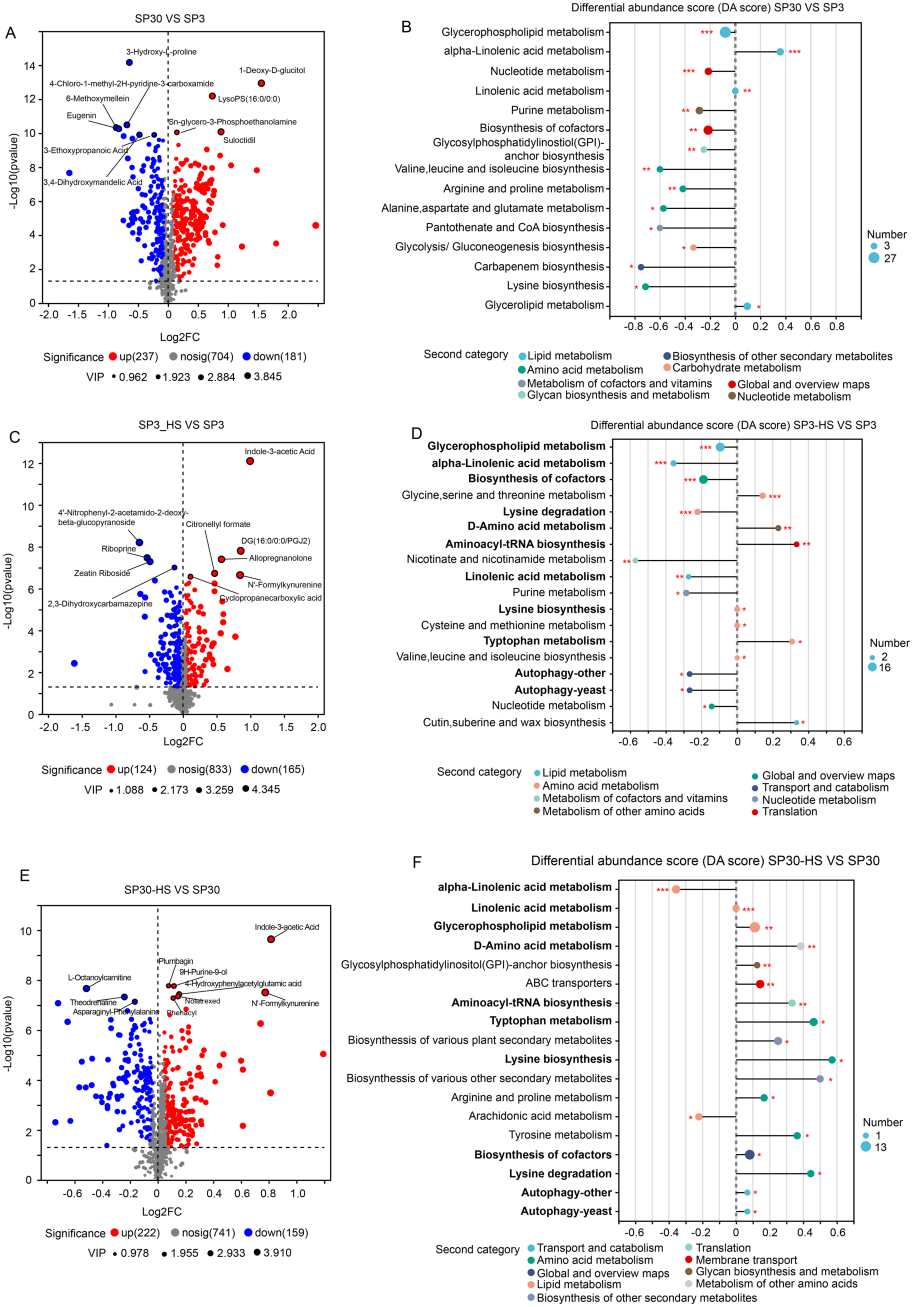
Differentially accumulated metabolites (DAMs) analysis of SP3 and SP30 under normal and HS conditions. **(A,B)** Volcano plot depicts the DAMs, and their KEGG enrichment analysis between the two strains under normal condition, respectively. **(C,D)** Volcano plot depicts the DAMs, and their KEGG enrichment analysis of SP3 under HS condition, respectively. **(E,F)** Volcano plot depicts the DAMs, and their KEGG enrichment analysis of SP30 under HS condition, respectively. For the volcano plot, the size of the points indicates the VIP value in the corresponding OPLS-DA model. For the differential abundance score analysis, the size of the point denotes the number of metabolites in the pathways. The length of the line represents the absolute value of DA score. Positive and negative DA scores represent the pathways tend to be up- and down-regulated, respectively.

With the volcano analysis, we found that 418 metabolites were differentially accumulated between monokaryon SP3 and SP30 (Figure 4A). Those DAMs were mainly enriched in the pathways of the “glycerophospholipid metabolism,” “alpha-linolenic acid metabolism,” “nucleotide metabolism,” etc. (Figure 4B). There were 124 metabolites that were up-regulated, and 181 metabolites that were down-regulated when SP3 was treated with heat (Figure 4C). These DAMs in SP3 were mainly enriched in the KEGG pathways of “glycerophospholipid metabolism,” “alpha-linolenic acid metabolism,” “biosynthesis of cofactors,” etc. (Figure 4D). As for SP30, 222 metabolites were up-regulated, and 159 metabolites were down-regulated under HS (Figure 4E). These DAMs in SP30 were largely enriched in the KEGG pathways of the “alpha-linolenic acid metabolism,” “linolenic acid metabolism,” “glycerophospholipid metabolism” as shown in Figure 4F. Interestingly, for SP3 most of the top 20 altered metabolic pathways tended to be down-regulated, whereas in SP30 those presented an opposite trend. Of the top 20 altered metabolic pathways, 11 pathways were the same, while the trends were different. It was worth to noting that the top changed pathway “glycerophospholipid metabolism” was significantly (*p* < 0.001) down-regulated in SP3, while was significantly up-regulated (*p* < 0.01) in SP30, highlighting the strain-specific differences of the metabolic profile in response to HS.

### 3.5 Glycerophospholipid metabolism pathway analysis

**Figure 5:**
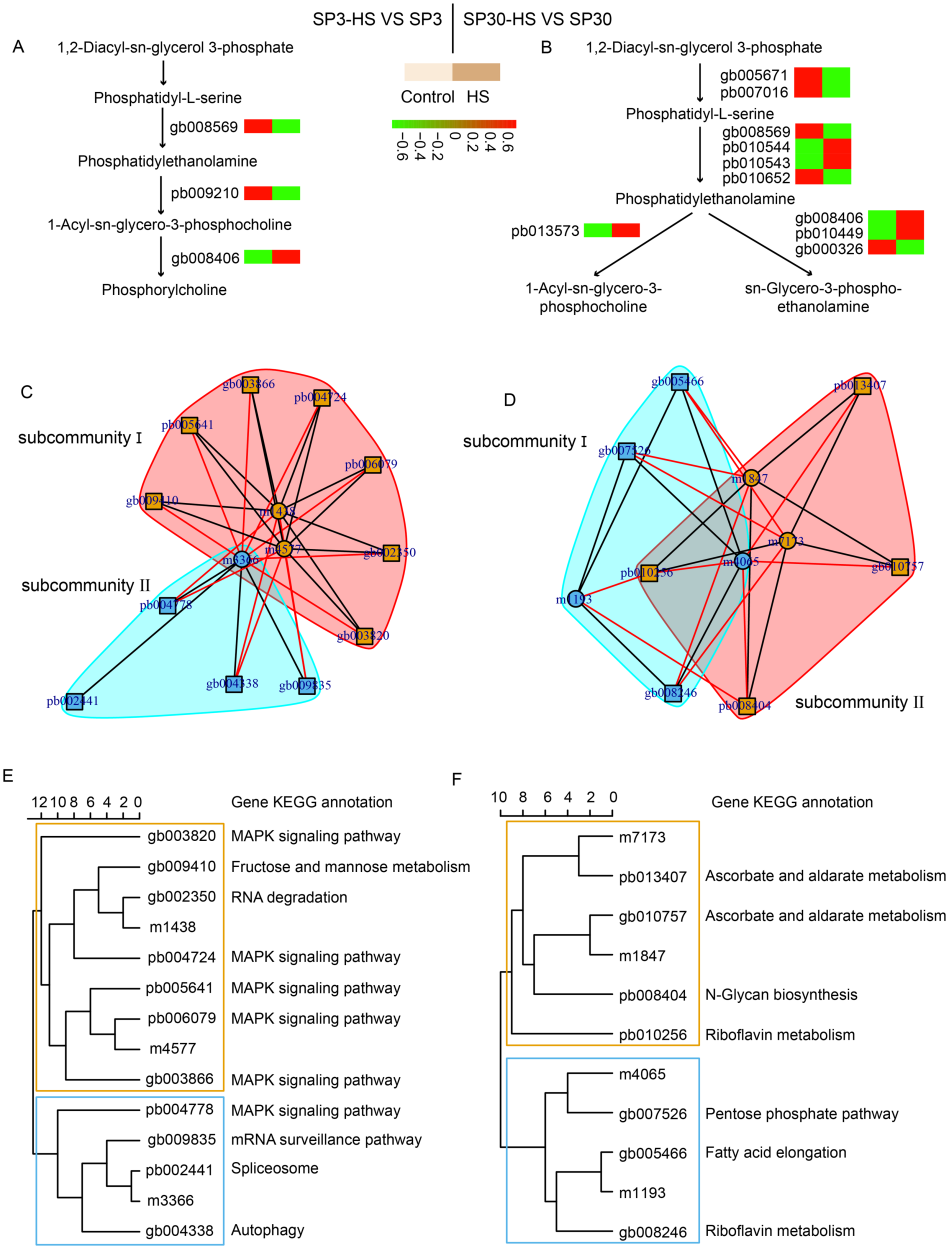
Putative modulation of glycerophospholipid metabolism pathway in SP3 and SP30 under heat stress. **(A,B)** Simplified representation of DEGs and DAMs in glycerophospholipid metabolism pathway. **(C–F)** Integration analysis of DAMs in glycerophospholipid metabolism pathway and DEGs using sPLS method (cut-off threshold 0.9).

Glycerophospholipid metabolism has been shown to be involved in heat stress responses in both plants (Sun et al., 2020; Feng et al., 2023; Zhou et al., 2024) and edible fungi (Yan et al., 2020; Jiang et al., 2022; Guo et al., 2023). The metabolomic data showed that 16 and 13 DAMs were detected in SP3 and SP30 under HS in the glycerophospholipid metabolism pathway, respectively (Supplementary Tables 6, 7), and the transcriptomics showed that only 3 and 10 DEGs were found in the pathway in SP3 and SP30 under HS, respectively (Supplementary Table 8). According to the KEGG pathway, we mapped those genes involved in the glycerophospholipid metabolism (Figures 5A,B). It showed that the glycerophospholipid metabolism was modulated differently in response to HS in the two monokaryons. Upon exposure to HS, only one gene was involved in the conversion of phosphatidyl -serine to phosphatidylethanolamine in SP3, whereas four genes were involved n SP30. The phosphatidylethanolamine was then catalyzed by different enzymes, which synthesized different downstream metabolites. To discover more DEGs which are putatively associated with regulation of glycerophospholipid metabolism, we conducted an association analysis using the DAMs detected in glycerophospholipid metabolism pathway and all DEGs. The arrow plot demonstrated a high agreement between the DAMs and DEGs datasets and the discriminative ability of each dataset (Supplementary Figure 2). Using the sPLS model, we were able to detect two highly associated communities (correlation coefficients ≥ 0.9 ) in the two monokaryons, respectively (Figure 5). The communities of SP3 consisted of 11 genes and 3 metabolites, with 2 metabolites were correlated with 7 genes in subcommunity I, and 1 metabolite was correlated with 4 genes in subcommunity II. As for SP30, 2 metabolites were correlated with 4 genes in subcommunity I, and 2 metabolites were correlated with 3 genes in subcommunity II (Figures 5C–F). For SP3, it was worth noticing that most of the highly associated genes were annotated as being involved in MAPK signaling pathways, which were completely different from what was observed in SP30 (Figures 5E,F).

### 3.6 Integrative analysis revealed networks between upregulated metabolites and regulatory genes

**Figure 6:**
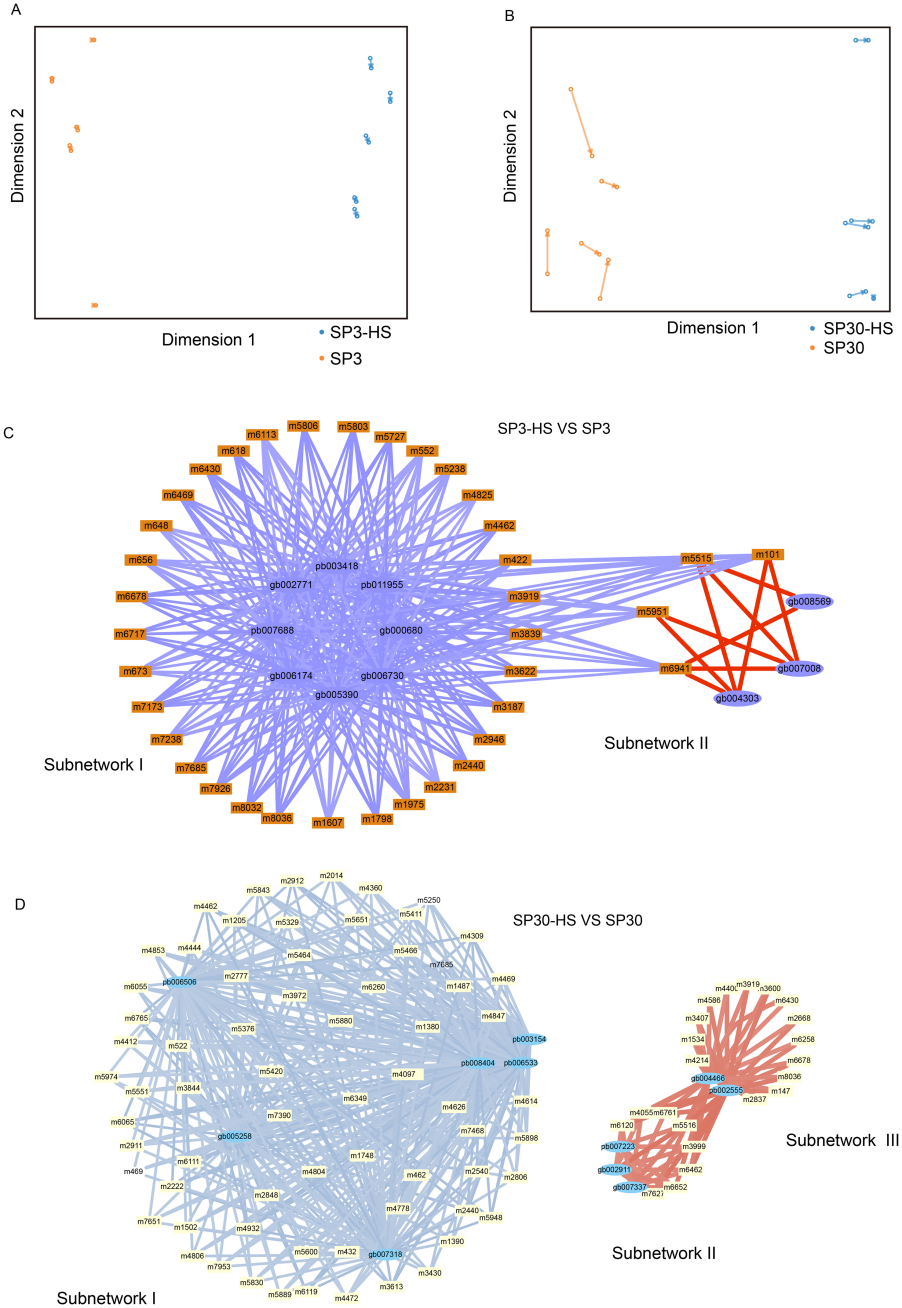
Transcriptome–metabolome wide association analysis of SP3 and SP30 under HS. **(A,B)** The sparse partial least squares (sPLS) association analysis between the upregulated metabolites and all DEGs. **(C,D)** The integration network of highly associated genes and metabolites at a threshold of 0.9. Positive correlations were represented by red edges, and negative correlations were represented by blue and light blue edges. The width of edges denote the correlation coefficients between nodes.

To unearth the gene regulatory network of the upregulated metabolites elicited by HS, an integrative analysis was performed using the upregulated metabolites with the DEGs of the two monokaryons. First, we examined the agreement between the upregulated metabolites and DEGs data sets using the sPLS regression model. The resulting arrow plots demonstrated a good association between the two datasets for both monokaryons (Figures 6A,B). All the identified highly associated DAMs and DEGs are presented in the Supplementary Figure 9. For strain SP3, the integration analysis revealed two distinct regulatory subnetworks in response to HS. The large subnetwork (subnetwork I) comprised 33 metabolites and 8 putative regulatory genes, and the subnetwork II comprised 4 metabolites and 3 putative regulatory genes. Interestingly, the metabolites in subnetwork I were all negatively correlated with genes, whereas in subnetwork II they were all positively correlated. The metabolites in subnetwork II were negatively correlated with genes in subnetwork I (Figure 6C). The top 5 hub genes included pb011955, pb003418, gb002771, pb007688, gb006174, and 3 of those genes were annotated to heat shock protein (Table 1). For SP30, 2 separated subnetworks were found, of which the smaller subnetworks were partitioned to 2 subcommunities (subnetwork II and subnetwork III). The subnetwork I included negatively correlated metabolites and DEGs, whereas the subnetworks II and III consisted of positively correlated metabolites and DEGs (Figure 6D). The top 5 hub genes were pb006533, pb003154, gb005258, gb007318, pb008404 in SP30, which were completely different from what discovered in SP3 (Table 1).

**Table 1:** The top 5 hub genes for strain SP3 and SP30 under heat stress.

SP3-HS VS SP3				
Gene ID	Log2FC	Padjust	KEGG	Swiss-Prot description
pb011955	3.015065	8.11E-205	Protein processing in endoplasmic reticulum	Heat shock protein 16
pb003418	2.197605	9.04E-178	Longevity regulating pathway	Heat shock protein 104
gb002771	3.061065	2.23E-101	Longevity regulating pathway	Heat shock protein 104
pb007688	1.614501	1.09E-134	Biotin metabolism	Biotin-protein ligase
gb006174	1.614501	1.09E-134	Biotin metabolism	Biotin-protein ligase
SP30-HS VS SP30				
pb006533	−5.35584	2.07E-241	-	
pb003154	−1.10127	1.19E-67	-	Uncharacterized secreted protein ARB_0804
gb005258	−6.18295	0	-	-
gb007318	−1.38438	1.03E-70	-	GMC oxidoreductase
pb008404	−1.24555	9.01E-170	Protein processing in endoplasmic reticulum	Probable mannosyl-oligosaccharide alpha-1,2-mannosidase 1B

Hub genes were identified by the number of edges adjacent to the node, i.e., node degree in the network.

## 4 Discussion

Unlike the sexual reproduction of plants and animals, of which the sperm and egg nuclei are fused to form a diploid zygote (2n), the haploid nuclei with different mating types, however, stay apart in fungal cells (n + n) (Nieuwenhuis and James, 2016). The constituent nuclei can, therefore, be recovered from the heterokaryons as haploid monokaryons, and the intact and original genome combination can be maintained forever. Therefore, in mushroom breeding sexually compatible haploid monokaryons are important materials in fungal breeding. Recent studies showed that the two separate nuclei function differently during development and interaction with the surrounding environmental factors (Gehrmann et al., 2018; Sperschneider et al., 2023; Shen et al., 2024). Therefore, it is crucial to uncover how the two genetically different nuclei handle environmental stimulus.

For *L. edodes*, heat resistance is one of the most important breeding goals, as high temperature events have frequently occurred in all production areas and severely reducing its productivity and fruiting body quality. The prerequisite for genetically improving its heat-resistance is to understand how shiitake mushrooms address the high temperature condition at the molecular levels. Though plenty of work has been done to elucidate the heat-resistance mechanism of shiitake, it is still far from being clear. Particularly, how the two physically separated monokaryons function in the heterokaryotic cell when confronted high temperature is still in its infancy. To the best of our knowledge, our work is the first pioneering study attempt to uncover the mechanism by which the two sexually compatible haploid nuclei handle heat stress.

Using the combination of the omics-based strategy and phenotypic parameters, we depicted the overall bare-bones how the two haploid nucleus tickled HS. Consistent with the findings in diploid mycelium, ROS production was elicited by high temperatures, which might contribute to the decrease of growth rate of mycelia. The varied extent of growth inhibition by HS suggested that the two monokaryons may have different capacity to resist HS. Further, the metabolome and transcriptome data supported the finding that the two nucleus SP3 and SP30 responded differently when challenged with high temperatures. We also found that SP30 has a greater number of DAMs and DEGs compared to SP3 under HS. The KEGG enrichment analysis showed that the DAMs for SP3 and SP30 were enriched in many different pathways. More interestingly, the enriched KEGG pathways for SP3 mostly tended to be downregulated, whereas those for SP30 showed an opposite trend. The glycerophospholipid metabolism pathways, which have been shown to be associated with heat-resistance in edible fungi (Yan et al., 2020; Jiang et al., 2022; Guo et al., 2023), were enriched in both strains in our study but with different regulatory mechanisms. These results can be a good complement to our previous findings, which described that the glycerophospholipid metabolism was markedly enriched in the heterokaryotic *L. edodes* mycelium in response to HS (Guo et al., 2023). Glycerophospholipids are the dominant molecules on cell membranes, conferring its stability, fluidity, and permeability. They are required for the function of membrane proteins, receptors, and ion channels and act as reservoirs for second messengers and their precursors (Hishikawa et al., 2014). Glycerophospholipids metabolism can be regulated by the MAPK signaling pathway. For example, the extracellular signal-regulated kinases (ERKs) and p38 MAPK kinases can regulate the activation of key enzymes in the glycerophospholipid metabolism pathway through phosphorylation (Johnson and Lapadat, 2002). MAPK signaling can also activate or inhibit some lipid metabolism-related transcription factors, such as PPAR*γ* (peroxisome proliferator-activated receptor γ). These transcription factors can regulate the expression of many genes involved in glycerophospholipid metabolism, thus indirectly affecting the synthesis, catabolism and transport of glycerophospholipids (Plotnikov et al., 2011). In return, the metabolism of glycerophospholipids can generate important second messengers, such as diacylglycerol (DAG), which can activate protein kinase C (PKC) and affect the activity of the MAPK signaling (Bill and Vines, 2020). As only a limited number of DEGs were found directly in the glycerophospholipid metabolism pathway, we performed an integrative analysis using the detected glycerophospholipid metabolites with all DEGs. We discovered that the MAPK signalling pathway was highly associated with the glycerophospholipid metabolism in SP3, which was not found in SP30. It suggests that for SP3 the MAPK signaling mediated glycerophospholipid metabolism reprogramming may function to tackle heat stress. Taking together, our results support the previous findings that the glycerophospholipid metabolism pathway is related to HS responses in fungi.

Thanks to the strong correlation between our metabolome and transcriptome dataset, we were able to construct a regulatory network of the DAMs and DEGs to delve deeper into the mechanisms by which the two monokaryons cope with HS. Our omics-integration analysis revealed distinct regulatory network structures between the two strains. The networks of the two monokaryons possessed completely different hub genes, further supporting our idea that the two monokaryons cope with HS using different strategies. In strain SP3, we found some highly correlated heat shock proteins (HSPs)that were not found in SP30. Two of the hub genes were annotated as HSP104. It can protect the protein from denaturation by interacting with HSP40 and HSP70 under high temperature. Knockout of the HSP104 gene causes the loss of tolerance to not only heat as well as the viability of cells stored at low temperatures (Tiwari et al., 2015). Another hub gene was annotated as HSP16. Such small HSPs are crucial for stabilizing cell membranes (De Maio and Hightower, 2021). Consequently, our results agreed with the importance of the role of HSPs in *L. edodes* heat-resistance found in dikaryotic mycelium (Wang et al., 2018; Wang et al., 2020; Ling et al., 2022), and further highlight the different regulatory mechanisms for the nuclei side by side in a cell.

## 5 Conclusion

Breeding for superior heat-resistant strains is critical for *L. edodes* production, since its cultivation is frequently threatened by high temperature stress. However, unlike plants and animals, the two sexually different nuclei stay side by side in cells and harbour different genetic structures and contribute differently during development. However, the mechanism by which the two nuclei deal with HS remains unclear. Here, we found for the first time that the two sexually compatible monokaryons regulate HS with different molecular machinery and provided informative findings from the aspect of metabolomics and transcriptomics. These results offer a holistic view of the nucleus-specific strategy for HS management, and provide candidate metabolites, responsive genes and regulatory pathways. Particularly, our results highlight the different regulation patterns of glycerophospholipid metabolism and HSPs in response to high temperature of the two nuclei of *L. edodes*. Nevertheless, these interesting findings should be subjected to further experimental validations.

## Data Availability

The datasets presented in this study can be found in online repositories. The names of the repository/repositories and accession number(s) can be found at: https://ngdc.cncb.ac.cn/gsub/submit/bioproject/subPRO046513/overview, PRJCA031400.
