# Supplementary figures and images for: Two sexually compatible monokaryons from a heterokaryotic Lentinula edodes strain respond differently to heat stress
Source: Front Microbiol. 2025 Feb 12;16:1522075. doi: 10.3389/fmicb.2025.1522075 (PMC11861359; doi:10.3389/fmicb.2025.1522075)

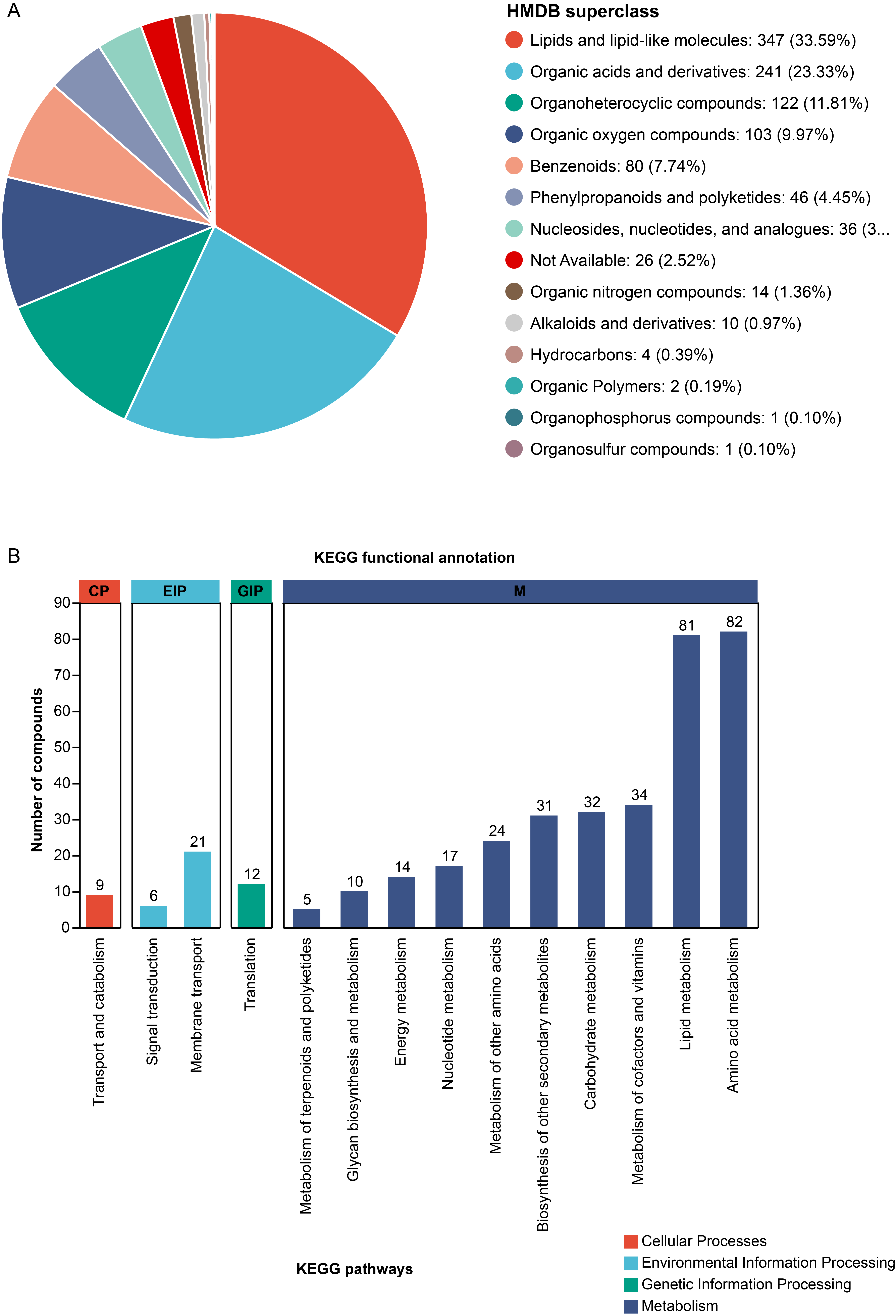

Supplement: Supplementary file 1 [file Data_Sheet_1.ZIP › Supplementary figure 1.tif]

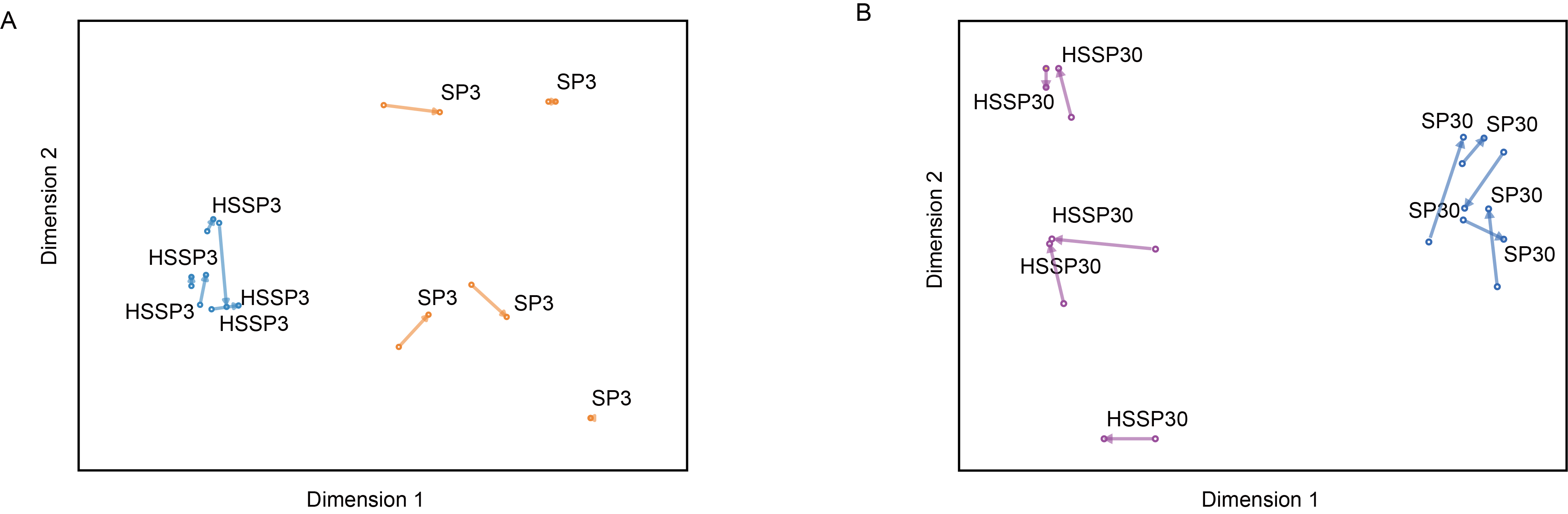

Supplement: Supplementary file 1 [file Data_Sheet_1.ZIP › Supplementary figure 2.tif]
